# Helicopter hoist operations in German mid-range mountains retrospective analysis of incidence, medical characteristics, and mission tactics

**DOI:** 10.1186/s13049-024-01297-9

**Published:** 2024-12-02

**Authors:** Sebastian Heinrich, Christoph Wielant, Frank Lerch, Mirko Friedrich, Jörg Braun, Florian Reifferscheid, Hans-Jörg Busch, for the Scientific Working Group of German Air Rescue

**Affiliations:** 1 Department of Anesthesiology and Critical Care, Medical Center - University of Freiburg, Faculty of Medicine, University of Freiburg, Hugstetter Str. 55, 79106 Freiburg, Germany; 2 German Air Rescue, DRF Stiftung Luftrettung Gemeinnützige AG, Rita-Maiburg-Str. 2, 70794 Filderstadt, Germany; 3 Department of Anesthesiology, Critical Care and Emergency Medicine, Artemed Clinics, St. Josefshospital Freiburg, Sautierstr. 1, 79104 Freiburg, Germany; 4 Department of Emergency Medicine, Faculty of Medicine, Medical Center - University of Freiburg, Sir- Hans-A-Krebs-Str., 79106 Freiburg, Germany; 5 Black Forest Mountain Rescue Service, Scheffelstr. 49, 79199 Kirchzarten, Germany; 6 Department of Anesthesiology and Intensive Care Medicine, University Medical Centre Schleswig-Holstein, Campus Kiel, Arnold-Heller Str. 3, 24105 Kiel, Germany

**Keywords:** Helicopter hoist operation, Mountain rescue, Hoist mission tactics, Low mountain ranges

## Abstract

**Background:**

Helicopter hoist operations (HHO) are an important option for rescue operations in rugged and challenging terrain. German mid-range mountains are characterized by the versatility of ground conditions, few urban structures, and frequent use for local leisure activities, including the practice of more or less high-risk outdoor sports. This retrospective analysis aims to investigate the incidence of rescue missions in German mid-range mountains requiring HHO. The contributing air rescue bases' operational tactics and the underlying medical characteristics, such as injury patterns and the provided medical care, are also reported.

**Methods:**

This study is a retrospective analysis of the documentation of HHO missions carried out at the air rescue bases in Freiburg, Nuremberg, and Bautzen staffed by emergency physicians between 01/2020 and 07/2022. Data was extracted from the German Air Rescue database. To assess the topics of interest, we conducted basic descriptive statistics.

**Results:**

Data selection retrieved 410 HHO-associated rescue missions. A total of 304 datasets, including HHO, were suitable for further statistical processing. Air rescue base Freiburg contributed 152, Nuremberg 63, and Bautzen 89 missions. HHO missions showed an increased frequency in the summer season and from Friday to Sunday. In this collective, 75% of the underlying diagnoses were trauma-associated; in 33% of all patients, traumatic injury of the pelvis or lower limb occurred. 28% of the patients were in a potential or actual life-threatening condition and were scored NACA 4 or higher. The rates of invasive medical treatment, such as endotracheal intubation (5%) or venous access (79%), were considerably higher than in overall emergency missions. In terms of mission tactics and cooperation with mountain rescue services, different approaches of the three air bases resulted in differences regarding first-on-scene rates and time spans.

**Conclusion:**

The results show a relevant year-round need to deploy emergency medical expertise to inaccessible terrain in the three regions examined. Detailed analysis showed relevant differences in operational tactics between the three bases and potential for optimization. Simultaneous alerting of the hoist helicopter and reliable and precise coordination with other rescue organizations involved, especially the local mountain rescue service and the rescue coordination center, can help to shorten both the treatment-free interval and the prehospital time for patients in inaccessible terrain.

*Trial registration*: The study is registered at DRKS (DRKS00033493).

## Background

One of the goals of establishing the air rescue medical service in Germany was to enhance the immediate care of severely injured patients and facilitate the swift transportation of injured patients to appropriate facilities [1, 2]. The scope of air rescue operations rapidly expanded to encompass the full range of emergency medical care and the transportation of patients needing intensive medical treatment [2–4]. In Germany, the air rescue medical service has become an integral part of the everyday emergency care structures and has been developed continuously [3–5]. The advancements in medical technology have expanded the possibilities for medical treatment and improved the capabilities of airborne operations and air rescue in Germany. Studies have highlighted their role in enhancing emergency treatment, primarily by reducing response and transportation times. This has led to an overall reduction in prehospital time [3–5]. The 2016 consensus paper on prehospital and hospital emergency care recommends planning the structure and emergency medical procedures in line with current guidelines. It advocates for a 60-min prehospital target time from alert to hospital admission for emergency patients [6]. It is often impossible to meet these targets for operations in rough or hard-to-reach terrain, resulting in patients not being transferred to the appropriate hospitals within the required timeframes, which can significantly worsen their prognosis. Helicopter hoist operations (HHO) are a well-established method of air rescue in coastal, marine, and alpine mountainous areas [7]. The potential benefits and demand for HHO in sub-alpine terrain have been previously simulated [8]. In recent years, the number of HHO-equipped air rescue bases in Germany has increased, especially to meet the demand for HHO in low- and mid-mountain ranges. The German low and mid-mountain ranges are characterized by diverse conditions, few urban structures, and frequent use of local leisure activities, including the practice of more or less risky outdoor sports. The main purpose of this analysis is to document the frequency of rescue missions in German mid-range mountains that require helicopter hoist operations (HHO). It also aims to examine the operational strategies of the participating air rescue bases, as well as the medical characteristics such as injury patterns and the medical care provided during these missions.

## Methods

**Fig. 1:**
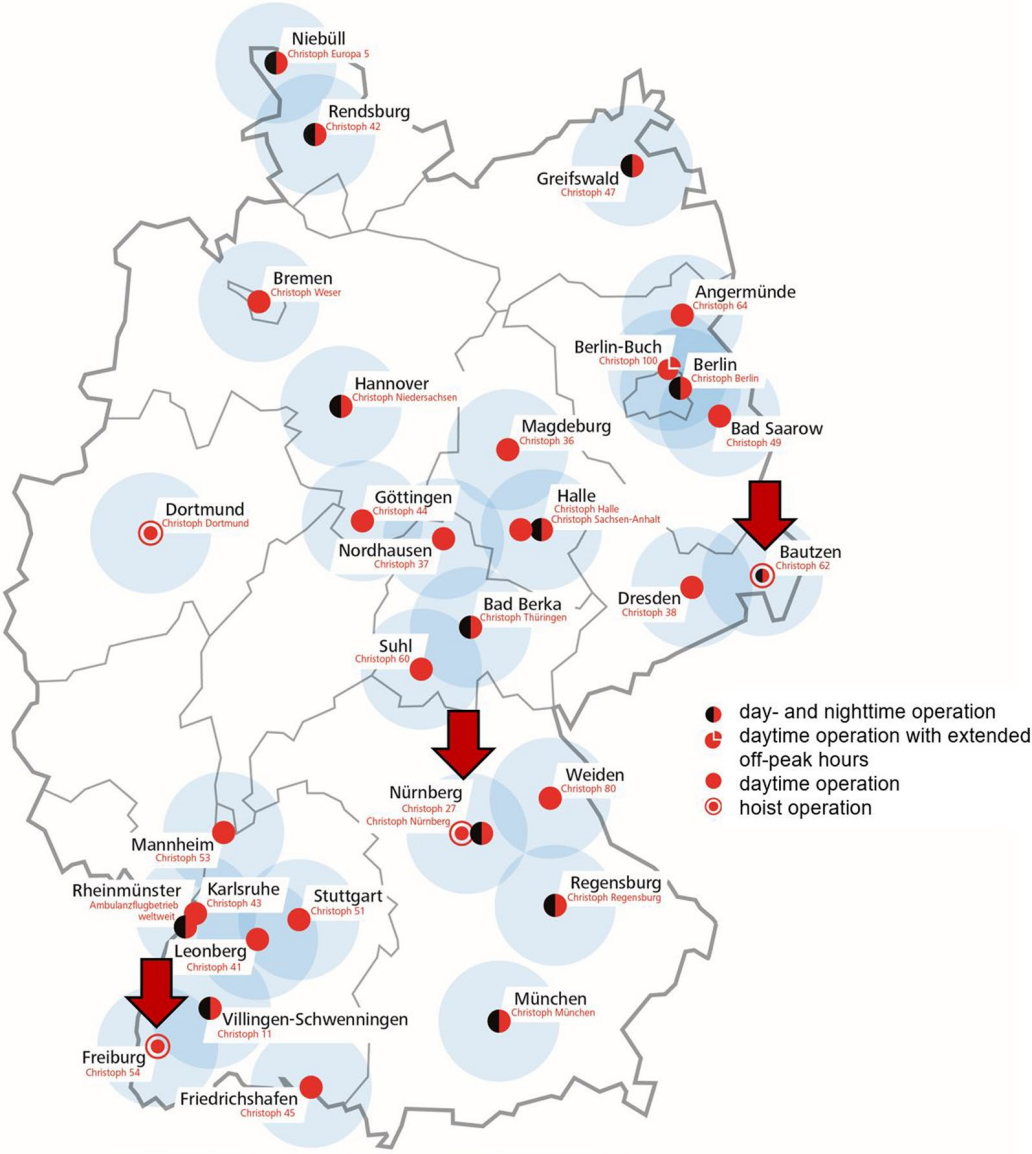
Map showing the geographical locations of the three air rescue bases with hoist operation addressed in this analysis

We retrospectively analyzed the routine documentation of the HHO-missions of the three air bases of the German Air Rescue, DRF Stiftung Luftrettung gemeinnützige AG Filderstadt, Germany, related to the mid-mountain ranges Black Forest (Freiburg), Franconian Switzerland (Nuremberg) and Saxony Switzerland (Bautzen) (Fig. 1). The helicopters are part of the public emergency service (EMS) and are alerted in addition to ambulances manned by paramedics. The helicopter crew consists of a pilot, a HEMS-TC (helicopter emergency medical system technical crewmember), and an emergency physician for daylight operation. All crew members are specially qualified and continuously trained for hoist operations. In an HHO mission, the individual roles are as follows: The pilot is flying the helicopter. The HEMS-TC is operating the hoist. The emergency physician is hoisted to the patient to provide further treatment. To assist the emergency physician and belay patient and physician in impassable terrain with the hazard of falling, a fourth crewmember of mountain rescue services qualified as rescue specialist helicopter (RSH) is added as required. The specific characteristics of the three bases, especially in cooperation with the rescue coordination center and the RSH crewmembers of the mountain rescue services, are presented in Table 1. All three air rescue bases are mandatorily alerted by the responsible local emergency dispatch centers. However, there are variations in how the hoist helicopters are dispatched. In Freiburg, the mountain rescue service, the emergency rescue service, and the hoist helicopter were usually alerted simultaneously based on the location of the incident or information from the emergency call suggesting that a hoist operation might be needed. In Nuremberg and Bautzen, the emergency response units were generally not alarmed in parallel but sequentially. First, the mountain rescue service and the emergency rescue service were alerted. The helicopter for hoist operations was only called in by the local rescue dispatch center when the mountain rescue service on-site or on the way to the scene determined that a hoist operation would be necessary.

**Table 1:** Characteristics and different approaches of the three air rescue base analysed in the present study

	Freiburg	Nuremberg	Bautzen
Call sign	Christoph 54	Christoph 27	Christoph 62
Helicopter	Airbus H 145	Airbus EC 135	Airbus H 145
Hoist configuration	Right side	Left side	Right side
Airside patient loading	Yes	No	Yes
Alerting of hoist helicopter (HH)	Primarily simultaneous alarming (HH, mountain rescue and emergency medical service) based on the information in the emergency call	Primarily sequential alarming. HH alerting is triggered by a mountain rescue service on-site or on their way to the scene	Primarily sequential alarming. HH alerting is triggered by a mountain rescue service on-site or on their way to the scene
Rescue specialist helicopter (RSH) pick up	Hoist missions in the vicinity: single hoist (EP) to the scene ahead of RSH pick-up if site is considered sufficiently safe. Longer approach routes or unsafe site: nearest available RSH pickup and transfer to the site	RSH picked up on-site or on the approach route. Drop-off (EP) on landing site nearby (weight and power reasons). Single hoist (RSH) to the patient, double hoist (patient, RSH) and transfer to landing site	RSH frequently already on scene or picked up on the approach route. With RSH on site, patient is prepared for hoisting ahead of the helicopter's arrival. If RSH is picked up on the approach route, double-hoist (EP, RSH) to the patient
Patient treatment & transfer to hospital	Patient treatment directly on scene, double hoist EP + patient lying in a vacuum rescue bag or in a sitting position using a rescue harness, airside patient loading, and direct transfer to hospital if beneficial	RSH and patient are double-hoisted from the scene and transferred to the landing site if patient can be hoisted without further treatment. Medical treatment (EP) at the landing site and loading if transport by helicopter is beneficial. Otherwise, transport by ambulance of the emergency medical service	Patient treatment directly on scene, double hoist EP + patient lying in a vacuum rescue bag or in a sitting position using a rescue harness, airside patient loading and direct transfer to hospital if beneficial

The operational data of all helicopters of the DRF Stiftung Luftrettung gemeinnützige AG (Filderstadt, Germany) is collected in a single database. Each mission, whether HHO or not, is documented in a standardized online form (HEMSDER-Database, Convexis, Germany). The documentation includes demographic data of patients, mission tactical data such as relevant timestamps and the medical treatment documentation in terms of diagnostics and therapy measurements. As the HHO capability of the air rescue base in Freiburg started in January 2020 and the documentation mode changed in August 2022, we included all HHO-related missions of the three air bases from January 2020 to July 2022. Mean and standard deviation and the Man-Whitney-U test were used to describe and compare continuous variables. The frequency and numerical proportion in percentage and Fisher-Exact-Test were used for categorical variables. The two-sided tests were considered statistically significant for *p* values ≤ 0.05. Statistical processing was performed using IBM SPSS Statistics 29 (Armonk, NY, USA). The institutional ethics committee of Albert-Ludwigs-University Freiburg approved the study (24-1063). The study is registered in the German Register of Clinical Studies DRKS (DRKS00033493).

## Results

**Fig. 2:**
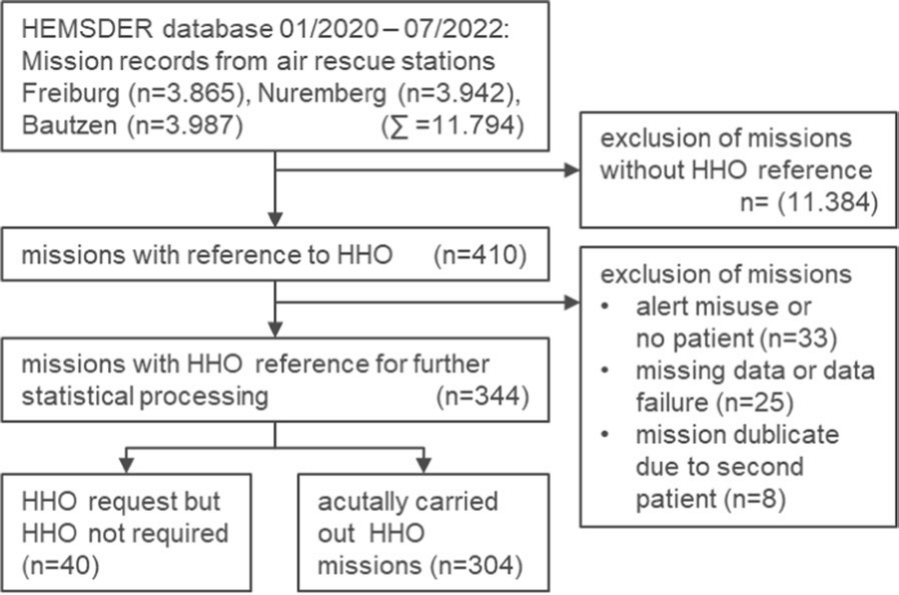
case selection according to Consolidated Standards of Reporting Trials (CONSORT) protocol

**Fig. 3:**
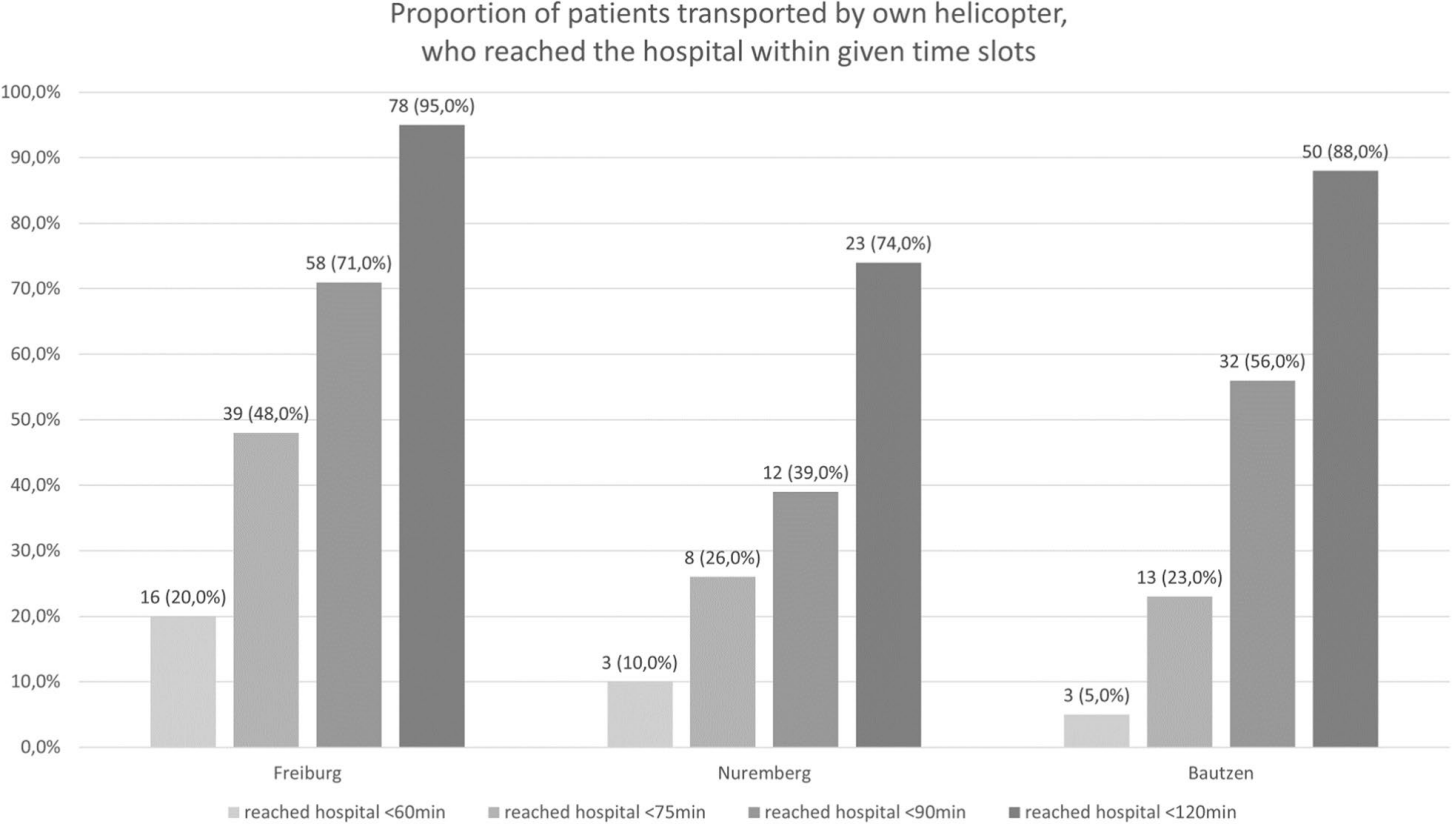
time slots in which patients reached the hospital

During the study period from January 2020 to July 2022, the air rescue bases in Freiburg, Nuremberg, and Bautzen carried out 11,794 missions. Of these, 344 cases were found to have a connection to HHO and were considered for further statistical analysis. Figure 2 shows the case selection according to the Consolidated Standards of Reporting Trials (CONSORT) protocol. Overall, 40 (12%) call-outs for HHO-missions were responded without performing HHO (Table 2). The lowest rate of HHO-call out without carrying out HHO was 4% in Bautzen, showing a statistically significant difference compared to 12% (Freiburg; *p* = 0.04) and 19% (Nuremberg; *p* = 0.003). Our register includes 304 HHO missions carried out by one of the three air bases (Freiburg n = 152; Nuremberg n = 63; Bautzen n = 89). No adverse safety events or injuries to bystanders, patients, or crew members were reported for any documented missions. Most HHO missions occurred in spring and summer (64%) and on weekends (58%). The time from alert to arrival on the scene was significantly shorter for the helicopter located in Freiburg (25 ± 13 min) compared to the helicopters located in Nuremberg (33 ± 21 min, *p* = 0.003) and Bautzen (40 ± 20 min, *p* = < 0.001). The first on-scene rate of the helicopter stationed in Freiburg (39%) is significantly higher compared to the other helicopters located in Nuremberg (11%; *p* < 0.001) and Bautzen (18%; *p* < 0.001). The overall mission duration was significantly shorter for the helicopter based in Freiburg (94 ± 36 min) compared to Nuremberg (118 ± 3 min; *p* < 0.001) and Bautzen (117 ± 37 min; *p* < 0.001). Figure 3 shows the proportion of patients who were transported by the HHO helicopter and reached the hospital in less than 60 min, 75 min, and 90 min after the callout of the mission. Pearson-Chi-Square testing revealed statistical significance for the 60-min interval (*p* = 0.04), the 75-min interval (*p* = 0.005), the 90-min interval (*p* = 0.006), and the 120-min interval (*p* = 0.007).

**Table 2:** Mission characteristics, data given as mean ± standard deviation or as stated

	Freiburg	Nuremberg	Bautzen	Overall
Missions with reference to helicopter hoist operations (HHO) [n / % of overall]	173/50%	78/23%	93/27%	344/100%
Carried out HHO missions [n/% of missions with HHO reference]	152/88%	63/81%	89/96%	304/88%
HHO requested but not carried out [n/% of missions with HHO reference]	21/12%	15/19%	4/4%	40/12%
HHO incidence [%, compared to all missions]	3.9%	1.6%	2.2%	2.6%
*Seasons and day of the week distribution*				
Spring (March–May) [n/%]	43/28%	24/38%	29/33%	96/32%
Summer (June- August) [n/%]	54/36%	23/37%	29/33%	106/35%
Autumn (September–November) [n/%]	32/21%	8/13%	21/24%	61/20%
Winter (December–February) [n/%]	23/15%	8/13%	10/11%	41/13%
Monday–Thursday [n/%]	66/45%	25/37%	36/40%	127/42%
Friday–Sunday [n/%]	86/55%	38/63%	53/60%	177/58%
*First on scene*				
First on scene: own helicopter [n/%]	60/39%	7/11%	16/18%	83/27%
First on scene: ambulance [n/%]	58/38%	30/48%	19/21%	107/35%
First on scene: mountain rescue [n/%]	27/18%	23/37%	52/58%	102/34%
First on scene: others [n/%]	7/5%	3/5%	2/2%	12/4%
*Mission times*				
Overall mission time [min]	94 ± 36	118 ± 38	117 ± 37	105 ± 32
Time from alert to arrival [min]	25 ± 13	33 ± 21	40 ± 20	31 ± 13
On scene time [min]	27 ± 16	35 ± 20	20 ± 14	26 ± 17
Time from arrival to handover in hospital [min] (only transports by own helicopter)	51 ± 18	60 ± 20	46 ± 20	51 ± 19
*Distances*				
Air rescue base to the scene [km]	27 ± 25	47 ± 36	39 ± 6	35 ± 26
Scene to hospital [km] (only transports by own helicopter)	21 ± 12	34 ± 23	28 ± 12	25 ± 16
*Patient transported by*				
Hoist helicopter [n/% of missions with HHO]	82/54%	31/49%	57/64%	170/56%
Ambulance with physician of hoist helicopter [n/% of missions with HHO]	8/5%	4/6%	7/8%	19/6%
Ambulance with other physician [n/% of missions with HHO]	2/1%	1/2%	0	3/1%
Ambulance without physician [n/% of missions with HHO]	46/30%	18/29%	19/21%	83/27%
Other helicopter [n/% of missions with HHO]	1/1%	3/5%	1/1%	5/2%
Others (private car, patient remains at the scene etc.) [n/% of missions with HHO]	13/9%	1/1%	5/6%	19/6%
*Medical procedures [n/% related to carried out HHO missions]*				
Airway Management and ventilation	10/7%	1/1%	3/3%	14/5%
Venous access	120/79%	53/84%	67/75%	240/79%
Cervical spine immobilization	44/29%	20/32%	18/20%	82/27%

The airline distances between the air rescue bases and their respective mission sites were shorter for the HHO missions near Freiburg (27 ± 25 km) compared with Nuremberg (47 ± 36 km; *p* < 0.001) and Bautzen (39 ± 6 km; *p* < 0.001). When the HHO helicopter transported the patient to a hospital, the airline distances between mission sites and the hospitals were shorter for Freiburg (21 ± 12 km) as well compared to Nuremberg (34 ± 23 km; *p* = 0.005) and Bautzen (28 ± 12 km; *p* = 0.002).

**Fig. 4:**
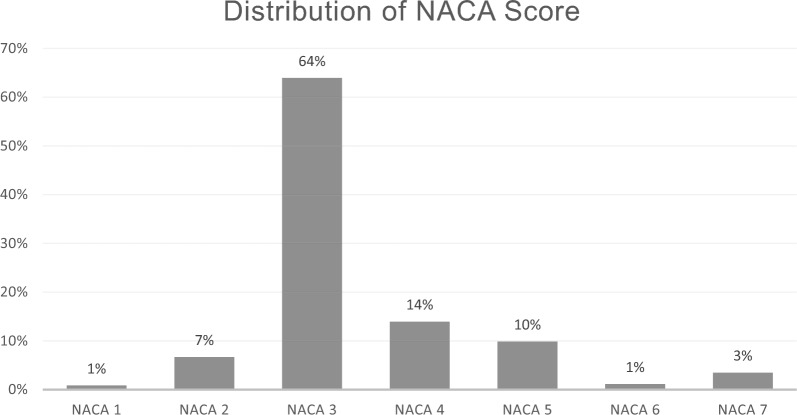
Distribution of National Advisory Committee for Aeronautics (NACA) Score

Table 3 presents the patients’ characteristics and the illness and injury patterns. The patients’ ages ranged from two years to 90 years, and the mean age was 48 ± 21 years; the differences between the three groups showed no statistical significance. Almost two-thirds of the patients (64%) were of male gender. The severity of illness or injury given as NACA-Score (National Advisory Committee for Aeronautics) shows that a cumulative 28% of the patients were in a potential or actual life-threatening condition and were scored NACA IV or higher. The detailed NACA distribution is shown in Fig. 4.

**Table 3:** Patient characteristics, data given as mean ± standard deviation or as stated

	Freiburg	Nuremberg	Bautzen	Overall
Male gender [n/%]	98/64%	41/65%	57/64%	196/64%
Age [years]	48 ± 21	43 ± 22	50 ± 20	48 ± 21
*Patterns of illness and leading injury*				
All traumatic injuries [n/%]	115/75%	53/85%	71/80%	239/79%
All non-traumatic causes [n/%]	37/25%	10/15%	18/20%	65/21%
Traumatic brain injury incl. cervical spine [n/%]	23/15%	8/13%	15/17%	46/15%
Chest trauma [n/%]	9/6%	7/11%	6/7%	22/7%
Upper limb injury [n/%]	26/17%	5/8%	11/12%	42/14%
Thoracic and lower spine injury [n/%]	8/5%	8/13%	4/4%	20/7%
Abdominal trauma [n/%]	5/3%	3/5%	0	8/3%
Pelvic and lower limb trauma [n/%]	44/29%	22/35%	35/39%	101/33%
Cardiac vascular diseases [n/%]	19/13%	1/2%	11/12%	31/10%
Neurological disorders [n/%]	10/7%	1/2%	2/2%	13/4%
Other causes (anaphylaxis, alcohol abuse, etc.) [n/%]	8/5%	8/13%	5/6%	21/7%

## Discussion

The results of our study indicate a year-round demand for HHO-capable helicopters in the three mid-range mountain regions we evaluated. Most HHO missions were conducted in spring and summer in all three regions. Especially in the region of Freiburg, where skiing is a common leisure activity in the winter months, the results might be influenced by the COVID-19 pandemic and the associated protective measures, which included a ban on the public operation of ski lifts in the winter of 2021. The accumulation of HHO missions on weekends reflects a solid connection to recreational activities such as hiking, climbing, mountain biking, and paragliding.

The disparity in the first-on-scene rate and the duration from alert to the arrival of the hoist helicopter must be seen in the context of the specific deployment areas and dispatching practices of each air rescue base. While the Freiburg air rescue base is frequently nearer to the HHO mission locations, the marginal difference in travel distances is insignificant compared to the helicopters' airspeed. It, therefore, does not account for the varying arrival times. Different alerting strategies are more likely to play a role in the hoist helicopter being the first rescue resource to arrive at the scene more frequently in missions around Freiburg. In Freiburg, the alarm for an HHO operation can be raised by the local emergency dispatch center primarily based on the information from the emergency call, such as geographical references matching with predefined regions where HHO is highly likely to be the only or fastest way to conduct a rescue operation. The HHO alarm can be activated without requiring other rescue services, such as the mountain rescue service, to be present on-site to request the HHO helicopter as a secondary measure. This also explains the lower rate of HHO requests fulfilled without HHO being conducted around the Bautzen air rescue base. The operational tactics in cooperation with the RSH of the mountain rescue service are essentially determined by the operational requirements. In most operations in the Freiburg area, which occur in wooded areas with no risk of falling, an emergency doctor can move around safely without being secured by the RSH. In these cases, the EP can be hoisted to the scene as the primary response to shorten the response time. If an RSH is necessary, it can be picked up and brought in secondarily to support the emergency physician. The deployment tactics must be adapted in steep or rocky terrain, where emergency physicians risk falling if improperly belayed. Deploying the RSH on scene, either primarily or together with the emergency physician, to ensure the on-scene safety of the mission has to be preferred in these situations.

For all three observed air rescue bases, HHO missions are rare, representing only 1.6% to 3.9% of the overall mission spectrum. This means crew members may go weeks or months without participating in an HHO mission. Due to this rarity, crew members must undergo continuous training to ensure safety and competence in HHO-related procedures. To supplement real flight training and enhance the frequency of training, indoor high-fidelity simulations are conducted by multiple operators and were also part of the annual training of the crews of this study [9, 10]. How each crew member can achieve the mandatory currency for HHO missions and increase individual proficiency should be part of further discussions.

In addition to the technical HHO-specific skills of all crew members, our results suggest that further requirements and competencies have to be addressed in crew training. It is not a standard task for EPs to provide care for patients, some of whom are seriously injured, without having assistance personnel in challenging environment and without protection from the weather with time being a relevant factor for patients’ outcome. Unlike EPs in ambulance or even helicopter service, who have access to the patient and can intervene any time during transport to the hospital, EPs involved in HHO-mission have to take into account that access to the patient and possibilities to intervene are limited during the hoist process. In addition, the EP must learn to assess the terrain and the corresponding dangers for him and his patient, as well as master rudimentary mountaineering safety techniques. The RSHs usually have no medical education and are unfamiliar with assisting medical interventions. However, in the HHO missions, they not only have to cover their main task of providing safety for patients and EPs on the scene but also of assisting the EP in live-saving interventions ahead of the hoist operation. An additional module of medical education and skill training in medical procedures assistance for the RSH crew members would address this issue.

Our data show that the majority of HHO missions are related to injured patients. While in our analysis, 79% of the HHO missions were trauma-related, the reference data of the physician-staffed ambulance service is only 20% [11]. Our findings are similar to those of a retrospective analysis of the HHO mission spectrum of two alpine-dominated air rescue bases [12]. Whereas air rescue bases in alpine terrain have a relevant amount of HHO-missions caused by uninjured or unharmed people, which ranges from 23% for daytime and 48% for nighttime HHO-missions, these missions were not relevant in our mid-mountain ranges collective [7, 12]. The cumulative proportion of patients with NACA score ≥ 4 in our cohort shows that a relevant part of the patients’ spectrum is severely harmed and needs EP treatment on scene. The number might even be higher as it is well known that EPs frequently underscore their patients, compared to an objective modified scoring system [13]. The frequencies of airway management (5% vs. 2%) and intravenous access (79% vs. 62%) in our cohort were higher compared to the reference data of the SQRBW for overall EP-staffed missions [11]. These findings and the high number of severely injured or otherwise harmed patients in our cohort underline the need for experienced and highly trained emergency physicians in the context of HHO, which has also shown to be beneficial in severe trauma patients [14].

### Limitations and lessons learned

Our study has limitations inherent in a retrospective analysis of routine documentation. As a detailed hoist protocol was not part of the routine documentation during the observation period, interesting aspects of HHO, such as the number of hoist cycles, incidence of airside patient loading, used hoist equipment like rescue bags, etc., have not been documented. To address this issue, the documentation module of HHO missions has been completely revised in 2022 and will undergo further revisions to cover the needs of further chart reviews. The comparability between the three air rescue bases is difficult in some aspects of this analysis. First, the characteristics of the three low mountain ranges and the corresponding mission spectrum do not entirely match. While in Freiburg, there are many operations in wooded and, therefore, difficult-to-access terrain, in Nuremberg and Bautzen, there are many steep rocky formations with injuries related to climbing sports. Secondarily, the Nuremberg air rescue base was limited in its HHO capabilities due to the operation of another type of helicopter, which did not allow airside patient loading. In 2023, the operator of the air rescue base in Nuremberg deployed an Airbus H145 analogous to the bases in Freiburg and Bautzen to lift these operational restrictions.

## Conclusions

Our results demonstrate a year-round necessity for HHO capability in the three mid-mountain ranges analyzed in this study. With nearly one-third of the patients having NACA score ≥ 4 and thus requiring urgent life-saving treatment, and a further amount of patients needing adequate analgesia, the need for adequately trained emergency physicians as integral parts of HHO capable crews is demonstrated. To reduce prehospital response time for patients, it is essential to dispatch HHO-capable helicopters early and accurately. The HHO-capable air base improves patient care by reducing response time, providing early physician-based treatment, minimizing pre-hospital time, and ensuring safe hoist operations. This is achieved by establishing binding commitments with local emergency dispatch centers, volunteer mountain rescue teams, and ground-based emergency rescue services. Our results also show that HHO missions are rare in the context of the overall number of missions. Therefore, conducting joint training sessions with mountain rescue services to maintain a high level of expertise and optimize collaboration appears beneficial.

## Data Availability

The data that supports the findings of this study are available from German air rescue service association (DRF e.V.) but restriction apply to the availability of this data, which were used under license for the current study, and so are not publicly available. Data are however available from the authors upon reasonable request and with permission of German air rescue service association (DRF e.V.).

## Abbreviations

EP: Emergency physician
EMS: Emergency medical service
CONSORT: Consolidated Standards of Reporting Trials
HEMS-TC: Helicopter emergency medical system technical crewmember
HHO: Helicopter hoist operation
NACA: National Advisory Committee for Aeronautics
RSH: Rescue specialist helicopter (degree of the mountain rescue service member)
